# Perspectives on the use of decellularized/devitalized and lyophilized human perinatal tissues for bone repair: Advantages and remaining challenges

**DOI:** 10.1016/j.mtbio.2024.101364

**Published:** 2024-11-26

**Authors:** Lauriana Solecki, Mathilde Fenelon, Halima Kerdjoudj, Roberta Di Pietro, Gianmarco Stati, Camille Gaudet, Eugenie Bertin, Jeremie Nallet, Aurélien Louvrier, Thomas Gualdi, Jessica Schiavi-Tritz, Florelle Gindraux

**Affiliations:** a CHU Besançon, Service d'Ophtalmologie, F-25000 Besançon, France; b Université de Franche-Comté, Laboratoire SINERGIES, F-25000 Besançon, France; c Hôpitaux Universitaires de Strasbourg, Service d'Ophtalmologie, F-67091 Strasbourg, France; d Université de Bordeaux, INSERM, BIOTIS, U1026, F-33000 Bordeaux, France; e Service de Chirurgie Orale, CHU Bordeaux, F-33076 Bordeaux, France; f Université de Reims Champagne Ardenne, Biomatériaux et Inflammation en Site Osseux (BIOS) EA 4691, F-51100 Reims, France; g Université de Reims Champagne Ardenne, Faculté Dentaire, F-51100 Reims, France; h Department of Medicine and Aging Sciences, G. d’Annunzio University of Chieti-Pescara, Chieti, Italy; i StemTeCh Group, Fondazione G. d’Annunzio, University of Chieti- Pescara, Chieti, Italy; j CHU Besançon, Service de chirurgie Maxillo-faciale, Stomatologie et Odontologie Hospitalière, F-25000 Besançon, France; k CHU Besançon, Service de chirurgie Pédiatrique, F-25000 Besançon, France; l CHU Besancon, Centre d’Investigation Clinique–Inserm CIC 1431, F 25000 Besançon, France; m University of Lorraine, CNRS, LRGP, F-54000 Nancy, France

**Keywords:** Perinatal derivatives, Amniotic membrane, Chorion, Wharton's jelly, Umbilical cord, Medical device, Placenta, Tissue graft, Regulation

## Abstract

Human amniotic membrane (hAM) has been extensively used for several decades as a bioactive scaffold for regenerative medicine. In its cryopreserved form—one of the main storage formats—the presence of viable cells has often been questioned. Furthermore, there is little published evidence of the role of endogenous amniotic cells from cryopreserved hAM in tissue repair.

Some technologies, often patented and combined, have facilitated the use of hAM. Decellularization and devitalization processes have been developed to ensure its safety and prevent immune rejection. Lyophilization and dehydration methods have had a significant impact on clinical practices by enabling storage at room temperature in the operating room and making handling and cutting easier. Consequently, the commercialization of hAM has expanded, initially in the USA, and now in Europe.

In the last decade, there has been growing interest in new perinatal tissues in clinical medicine. Similar processes have been adapted for these tissues to prevent immune or inflammatory reactions, and to improve storage and make them easier to use. For example, in the USA, many products marketed for wound healing undergo lyophilization, sometimes in combination with decellularization.

Given our expertise, we wanted to highlight the potential of decellularized/devitalized and lyophilized perinatal tissues in regenerative medicine, particularly for bone repair. In this opinion paper, we discuss why these tissues represent the future of regenerative medicine, their potential drawbacks and strategies to overcome these challenges.

## Graphical abstract

Our own process applied to perinatal tissues (adapted from Refs. [17,18,21,28–30,165]). Abbreviations: hAM: human amniotic membrane, hACM: human amnio-chorionic membrane, hUC: human umbilical cord, hUC-AM: human umbilical cord amniotic membrane, hUC-WJ: human umbilical cord Wharton's jelly.

**Figure:**
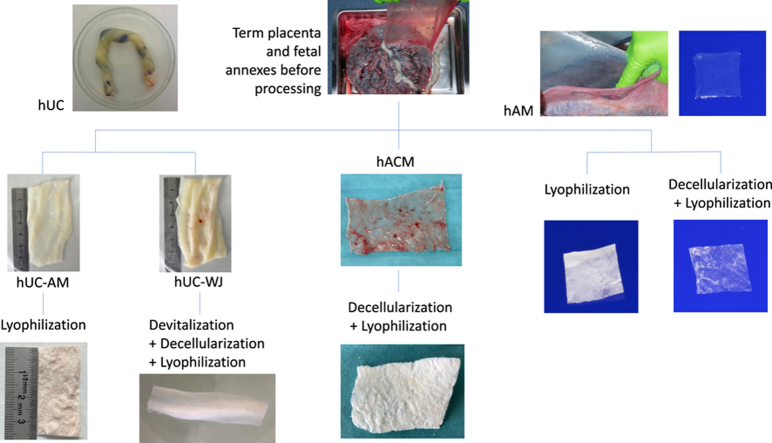

## 1 Introduction

The term “perinatal” refers to birth-associated tissues that are obtained from term placentas and fetal annexes, and more specifically refers to the amniotic membrane, chorionic membrane, chorionic villi, umbilical cord (including Wharton's jelly), the basal plate (including maternal and fetal cells), and the amniotic fluid [1].

Among perinatal tissues, the human amniotic membrane (hAM) was the first allograft used clinically and is now widely used to treat ocular surface disorders and improve wound healing [[2], [3], [4], [5], [6], [7]].

Currently, in France and other European countries, the clinical application of hAM for homologous use (the same essential function in the recipient as in the donor) is only routinely allowed for ophthalmic purposes. Other indications are restricted and require approval for clinical trials or compassionate use. In Germany, for example, hAM is authorized for use in orbital, oral and maxillofacial surgery, gynecological surgery, and as a temporary skin substitute [8]. In the USA, FDA-approved uses of hAM include wounds, ulcers, burns, adhesions, and skin injuries. It is also widely used in surgical fields such as gynecology, plastic surgery, gastrointestinal surgery, traumatology, neurosurgery, and ophthalmology [7,9].

Given our team's research focus on tissue regeneration, particularly bone repair, we have published several review studies [[10], [11], [12], [13], [14], [15]] and explored the potential use of hAM as a bioactive membrane in experimental/pre-clinical models and clinical applications [[16], [17], [18], [19], [20], [21], [22], [23], [24]]. Over the last decade, given the growing interest in perinatal derivatives (PnD) [1] and the increasing clinical use of perinatal tissues [25], we have also conducted experimental research in this field [[26], [27], [28], [29], [30]]. Our involvement in the European Cooperation in Science and Technology (COST) SPRINT community has led to significant andrecognized advances in the PnD field [25,[31], [32], [33], [34], [35]].

Given our expertise, we wanted to share our insights on the use of decellularized/devitalized and lyophilized perinatal tissues as biomaterials for bone regenerative medicine in this opinion paper.

### 1.1 Human amniotic membrane: the most widely used perinatal tissue

The hAM, which surrounds the amniotic cavity and the amniotic fluid, is composed of a single layer of amniotic epithelial cells (hAEC), a basement membrane and an avascular stroma. This stroma is populated by human amniotic mesenchymal stromal cells (hAMSC) and is underlain by the human chorion (hC) [36]. Its thickness varies between individuals and depends on the location from which the sample is taken, typically from 70 to 180 μm [37,38].

As an allograft, hAM was first used in clinical practice in the 1900s, primarily to treat ocular surface disorders and to enhance wound healing [[2], [3], [4], [5]]. Over the following decades, its application extended to other therapeutic areas, including gynecology, oral and maxillofacial surgery, orthopedics, neurology, urology and oncology. A recent review provides a comprehensive overview of its various therapeutic targets and its mechanisms of action [7]. Additionally, hAM has been widely used as a matrix in regenerative tissue engineering, including in combined constructs [6,15].

The beneficial effects of hAM have been extensively described in the literature. It serves as a biocompatible scaffold with suitable mechanical properties (permeability, stability, elasticity, flexibility, resorbability and transparency) [14,37]. It has antifibrotic [39], anti-scarring [40], antibacterial [40], antiviral [40], antimicrobial [41], and anti-adhesive effects in wound healing [40], along with anti-inflammatory [42,43] and analgesic properties [[44], [45], [46]]. hAM is known to modulate angiogenesis, with both pro- and anti-angiogenic properties [40,47]. It induces epithelialization, supports cell adhesion and growth [48], and promotes wound healing [40,47]. Finally, it has been recognized for its anticancer properties with low tumorigenicity [49,50] and low immunogenicity [51], making it highly suitable as an allograft/scaffold. These therapeutic effects are primarily attributed to the release of macromolecules including growth factors. However, there is still no clear evidence about the role of the endogenous amniotic cells in tissue repair. A recent review [6] provides a comprehensive overview of the growth factors and cytokines contributed by hAM, including basic fibroblast growth factor (bFGF), epidermal growth factor (EGF), hepatocyte growth factor (HGF), keratinocyte growth factor (KGF), nerve growth factor (NGF), transforming growth factor (TGF)β, interleukins (IL) 4, 6, 8, 10 and tissue inhibitors of metalloproteinases (TIMPs) 1, 2, 4.

#### 1.1.1 hAM immunogenicity, host response and degradation

hAM is widely regarded as an immunologically privileged allograft, meaning it has a lower likelihood of inducing an immune response when transplanted. However, there is only limited scientific evidence of this. In the 1980s, Akle et al. found no anti-HLA antibodies in the serum of four volunteers who had received a subcutaneous graft of fresh hAM [52]. They investigated the expression of HLA Ags in hAM and did not detect HLA class I (A, B, C) or II Ags using immunofluorescence on freshly collected or cultured hAEC. However, weak (or low) HLA Ag expression was detected when more sensitive radiobiological techniques were used [53]. In the following decade, Akle's results were challenged by the scientific community who focused on HLA detection from whole amnion or isolated cells [[54], [55], [56], [57]]. In 2001, Kubo et al. identified in a xenotransplantation model, immunoregulatory factors within cryopreserved hAM such as HLA-G and Fas ligand, which have been linked to its low immunogenicity [51].

Clinically, no immune reaction has been described after apposition or grafting of fresh hAM in wound healing [2,3,5], ocular surface reconstruction [58], or oral surgery [59,60]. Similarly, neither frozen [5] nor cryopreserved hAM [22,61,62] produced an excessive immune response or foreign body reaction when grafted in oral mucosal, on corneal defects or venous ulcers in clinical cases. So, we postulated that neither the hAM grafting site (with more or less immune privilege like the cornea) nor its grafting format (with more or less viable cells remaining) seem to influence its immunogenicity. These good clinical outcomes seem to be attributed to the hAM's purported low immunogenicity due to a lack of HLA class II antigens [51,63].

Over the years, this allograft has been associated with low immunogenicity and a high degree of clinical safety given the low rejection rate for a large number of transplants: 22 776 units in Europe between January 01, 2022 and December 31, 2022 [64]. Within the COST SPRINT, we detailed its effects on both inflammation and immune response mechanisms [31].

In a subcutaneous animal model, our *in vivo* studies have shown that neither fresh hAM nor preconditioned hAM (i.e., tissue cultivated in control medium or osteogenic medium for three weeks) induced an acute inflammatory reaction over eight weeks, compared to the control (human skin) [19]. Its degradation was slow compared to the human skin control. A slight difference in tissue degradation was observed between fresh/non-osteodifferentiated hAM and hAM cultivated in osteogenic medium, likely due to the presence of minerals.

In a similar animal model, we observed that fresh hAM that was cryopreserved and lyophilized without decellularization is a biocompatible matrix, inducing a slight-to-moderate reaction compared to sham-operated control samples [18]. However, the reaction was more pronounced for decellularized and lyophilized hAM two months after surgical implantation, probably due to its remnants. Because host cell infiltration occurred later in this specific hAM format, we contend that it has a protective effect against cellular infiltration like that offered by the barrier membrane used in clinical practice.

We studied the resorption of hAM in oral surgery, differentiating between implanted hAM and hAM used as a covering [22]. But we found it challenging to analyze its degradation because it is not directly visible after being implanted. Some authors reported complete histological resorption of cryopreserved hAM after 4 weeks in preclinical animal studies [22]. Others suggested that hAM has excellent compatibility with bone grafts, demonstrating good containment of the material and resorption without the formation of voids or detritus [22]. When used as a cover, lyophilized or dried amnion disappeared within 7–10 days in clinical settings, with complete resorption occurring after 2 weeks [22]. In contrast, full resorption was slower (3 weeks) when using fresh or glycerol-preserved hAM. Additionally, animal studies revealed complete resorption of cryopreserved hAM after 2 weeks or after 5 weeks when multilayered [22]. Lyophilized hAM achieved faster epithelialization (3 weeks) compared to cryopreserved (4 weeks) or dried hAM (6 weeks) [22]. Achieving a balance between degradation and epithelialization is a key clinical objective.

#### 1.1.2 hAM mapping

In the last decade, there has been growing interest in mapping of the hAM [65,66]. Recently, we reported that hAEC or hAM itself from amniotic sub-regions differ considerably in their morphology and functional properties and their content/release of bioactive factors [38,65,67], therefore impacting its osteogenic potential [65]. We observed that hAEC from the intermediate subregion of hAM was more prone to differentiate *in vitro* towards an osteogenic phenotype than hAEC from other subregions [65]. On the contrary, no variations were observed in both the expression of HGF, KGF and NGF in different zones of fresh hAM [68], and the total amount of soluble proteins in DMEM/glycerol cryopreserved hAM [69].

The mechanical properties might also vary depending on how far the subregion is located from the placental disk. In fact, some studies showed that hAM adjacent to the placental disk were significantly thicker and stronger compared to distal samples [38,70].

To our knowledge, no study has investigated the potential impact of hAM subregions on healing, including bone repair. Moreover, although hAM mapping seems to be relevant, it has yet to be implemented in tissue banking. Similarly, there are no standards for cell survival or potential, growth factor release in hAM patches despite intra or inter-donor variability. The clinical efficacy of hAM patches may not be comparable when used as graft given the different composition.

In this context, the effect of cryopreserved hAM harvested from different donors on the efficiency of wound healing has recently been studied. The aim was to understand whether inter-placental variations are critical in the wound healing rate or if they are suppressed by the processing/application chain and the patient's status at the time of application [71]. The mean efficiency for each placenta, expressed as an average of wound area reduction (%) 7 days after the hAM application (baseline, 100 %), was calculated from at least 10 applications. No statistical difference between the nine placentas' was found in the progressive phase of wound healing. The authors suggested that if there are intra- and inter-placental differences in healing efficacy of hAM sheets, they are overridden by the current health status of the subject or even the condition of individual wounds. Hopefully, enriched populations derived from different hAM sub-regions could exert more evident and consistent effects.

#### 1.1.3 hAM storage

The usual storage formats for hAM include cryopreservation, lyophilization or air-drying (dehydration) [72,73]. While cryopreservation requires a cryoprotectant, lyophilization or freeze-drying involves removing water from hAM through sublimation. During air-drying or dehydration, the hAM is kept at room temperature under a laminar flow hood and exposed to air for different time periods. Lyophilization and air-drying are generally followed by gamma irradiation (sterilization), and sometimes referred to as “hyperdried hAM” [[74], [75], [76], [77], [78], [79]]. It appears that freeze-drying does not affect the biological structure or the cytokine content of hAM [80]. In contrast, gamma irradiation significantly decreased TIMP-4, bFGF and EGF, and caused structural damage to the epithelium, basement membrane and lamina densa [80]. Most of these treatments were developed specifically for hAM storage and have been patented by academic labs and companies [81].

Previous studies on fresh or cryopreserved hAM have found trophic effects without cell migration from the hAM to the damaged tissue [39,82,83]. Additionally, the survival of amniotic cells after hAM cryopreservation is disputed [17,84,85]. We recently questioned the role of residual cells in tissue repair and the relevance of evaluating their viability, given that hAM primarily functions as a reservoir/matrix rich in growth factors and cytokines [86]. Our viewpoint appears to be supported by the scientific community [73].

Contrary to cryopreservation, cells do not survive freeze-drying or air-drying due to removal of water. The use of lyoprotectants such as trehalose [15] could theoretically circumvent this issue [73]. Because water loss from lyophilization may affect the physical and biological structures of amnion, trehalose can replace some of the water content in the cells, potentially stabilizing proteins and other components [87]. Both processes may impact the protein content, although it is less likely with cryopreservation [73]. Along these lines, it has been shown that dehydration has a significant effect on the total growth factor and cytokine content [88]. However, freeze-drying or air-drying also has important advantages. A primary benefit is modification of the tissue's mechanical properties, allowing the scaffold to be cut easily to the desired size and shape during surgery [76,89]. Another advantage is that the hAM can be preserved at room temperature for extended periods without degradation, simplifying its transport and storage, and thus reducing the product's costs [48,89]. Finally, lyophilized and (hyper)dried hAM are immediately useable after rehydration, while cryopreserved hAM requires about an hour for thawing and approximately 15 min of rinsing prior to use.

Recently, lyophilized hAM was reported to be more efficient in managing pterygium compared to cryopreserved hAM, although the authors did not explain why [90,91]. It appears they did a trehalose pre-treatment, but there is no mention of it in the product description on the tissue bank's website. So, the benefit of lyophilized hAM is difficult to authenticate and may be linked to lyoprotectant use.

In an interesting hAM mapping study, cryopreservation using DMEM/glycerol was ideal for preserving the structural integrity and soluble protein content in both placental amnion and reflected amnion hAM subregions [69].

Thus, each preservation method has its advantages and disadvantages. Recently, Hofmann et al. [73] summarized the advantages and disadvantages of the various graft preparation, preservation and storage techniques. Jafari et al. [92] stated that identifying a preservation method that effectively safeguards hAM properties remains an ongoing endeavor particularly considering its histological, physical, and biochemical characteristics.

#### 1.1.4 hAM de-epithelialization, “full” decellularization or devitalization

Additional enzymatic, chemical, or mechanical treatments have been applied to hAM such as *de-epithelialization*, with the removal of epithelial layer/cells to improve epithelial recipient cell migration and proliferation [93,94] or *“full” decellularization* with the removal of all amniotic cells to prevent the risk of rejection [95,96].

For years, the de-epithelialized (also called denuded) cryopreserved format has been the preferred choice for ocular surface reconstruction [93,94]. We find that many authors refer to a “decellularization” or “acellular” process when they actually performed “de-epithelialization”. To make it easier for the reader, in the following section, we differentiate between the two formats by adding the term “full” to the “decellularization” designation.

Decellularization results in a significant decrease in hAM thickness. Alteration of its ultimate tensile strength, extensibility, or elasticity, and its growth factor content is controversial [73,95]. The term “acellular hAM” is also often used in the literature. “Decellular” or “acellular” refer to a biological material lacking intact cells/nuclei or the ability to extract cells [97]. Decellularized hAM was manufactured to avoid sensitization and immunological responses, as well as to reduce the risk of infection and the high expenses associated with the use of living cell-containing grafts [98]. Many materials and methods have been widely used for this aim [97].

Both de-epithelization and decellularization strategies could be applied to fresh and cryo-preserved hAM, but they are mainly combined with lyophilization or air-drying, and mostly used for tissue engineering purposes [15]. A study evaluated the impact of decellularization based on the hAM's proteome profile, physicochemical features, as well as the attachment, viability, and proliferation of umbilical cord-derived MSC (hUC-MSC). They reported that both sides of decellularized hAM supported the growth and proliferation of hUC-MSC. Interestingly, comparative studies showed that the basal side of decellularized hAM produces higher hUC-MSC proliferation than the epithelial side [99].

We compared how preservation methods affected hAM properties for tissue engineering applications [18]. Histological staining revealed that the decellularized and lyophilized hAM architecture was disrupted, and that it was 84 % thinner than fresh hAM (p < 0.001). Type IV and type V collagen, elastin and laminin were preserved on decellularized and lyophilized hAM. Additionally, maximal force before rupture of decellularized and lyophilized hAM was 92 % higher than cryopreserved hAM and lyophilized hAM (p < 0.01), while decellularized and lyophilized hAM was 37 % more stretchable than fresh hAM (p < 0.05). Finally, decellularized and lyophilized hAM was the most suitable scaffold for human bone marrow MSC proliferation.

Moreover, the devitalization process has notable advantages [100]. The physical processing consists of freeze–thaw cycles or freezer-milling that disrupts cellular and nuclear membranes but may not fully remove DNA moieties, cell-associated proteins, and other cell remnants. We have found that some publications confuse vitality with residual activity and viability related to living cells [73].

Most of these treatments (de-epithelialization, full decellularization or devitalization) have been patented by academic labs and companies [81]. After a decellularization/devitalization process, residual DNA content, cell-associated proteins, and other cell debris may still be present [101]. While these components are known to be immunogenic, they are rarely measured or quantified in hAM used clinically and/or commercialized. This is mainly due to the fact that this allograft does not cause any immune reaction as seen above. DNA quantification could help to better characterize the process and standardize the product's quality, given the increasing use of decellularized/devitalized hAM in tissue engineering [15,102] and new multilayer composites in recent years [103].

The control over full decellularization/devitalization processes is even more important in Europe, as different sets of rules can apply at the same time. First, hAM decellularization or devitalization is not currently addressed in the current “Guide to the Quality and Safety of Tissues and Cells for Human Application” (5th Edition) [104]. Second, as mentioned in the article 2 of the new Regulation on Standards of Quality and Safety for Substances of Human Origin (SoHO) Intended for Human Application [105], tissues of human origin containing non-viable cells or rendered non-viable now fall into two regulations: “Where non-viable SoHO or their derivatives, within the meaning of Article 2, points (16) and (17), of Regulation (EU) 2017/745 [106], incorporate, as an integral part, a medical device, and where the action of the non-viable SoHO or their derivatives is principal to that of the medical device, this Regulation applies to the non-viable SoHO or their derivatives and the final combination shall be subject to this Regulation (EU) 2024/1938 [105]. Where the action of the non-viable SoHO or their derivatives is ancillary to that of the medical device, this Regulation applies to all SoHO activities to which the non-viable SoHO or their derivatives are subjected up to and including their distribution for integration into the medical device, and the final combination shall be subject to Regulation (EU) 2017/745 [106].” Third, decellularization and devitalization do not belong in non-substantial modifications as listed in Regulation 1394/2007 on advanced therapy medicinal products [107]. On the contrary, the decellularization process is considered a minimal manipulation in the FDA's Regulatory Considerations for Human Cells, Tissues, and Cellular and Tissue-Based Products [108]. This raises the question of which of these specifications apply to the product and are necessary to fully characterize the entire process.

#### 1.1.5 Additional treatments

It is noteworthy that viral inactivation by chemical treatment coupled with drastic microbiological and endotoxin controls allows for hAM collection after vaginal delivery for one commercial product (Visio Amtrix®, Horus Pharma: a freeze-dried and gamma-sterilization hAM [[109], [110], [111]]). Because of these processes and controls, and to circumvent the low number of caesareans in some hospitals/countries, the collection of placenta from vaginal delivery could increase the number of banked hAM.

### 1.2 From human amniotic membrane to other perinatal tissues

**Fig. 1:**
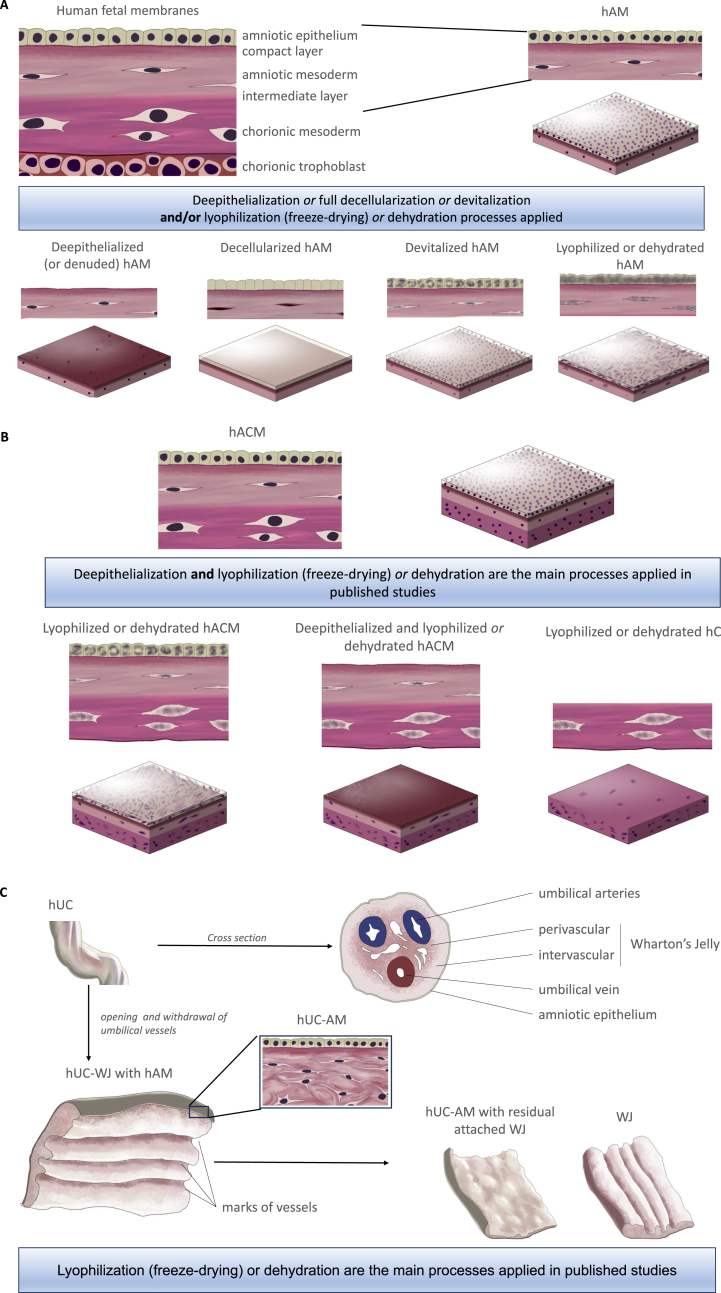
Process typically applied to perinatal tissues. (A) hAM, (B) hACM and (C) hUC. Abbreviations: hAM: human amniotic membrane, hACM: human amnio-chorionic membrane, hC: human chorion, hUC: human umbilical cord, hUC-AM: human umbilical cord amniotic membrane, hUC-WJ: human umbilical cord Wharton's jelly.

During the last decade, new PnD [1,32] such as hC, human amnio-chorionic membrane (hACM), umbilical cord (hUC), umbilical cord amniotic membrane (hUC-AM), umbilical cord vascular region (hUC-V), or umbilical cord Wharton's jelly (hUC-WJ) have been tested clinically and/or been introduced commercially [25,34,112]. The most commonly used new perinatal tissues are described below and depicted in Fig. 1 (A, B, C).

#### 1.2.1 Human chorion and amnio-chorionic membrane

hC and hACM have been widely used in oral and periodontal surgery because they are thicker than hAM [13,22].

Decellularization processes have been developed for both tissues to increase product safety due to likely contamination by the maternal cells on hC [113,114]. But surprisingly, most products have not undergone a decellularization process [7,9,13,22,81,115,116]. In oral and periodontal surgery, hC and hACM are mainly lyophilized or dehydrated and often subjected to a de-epithelialization process [13,22]. To our knowledge, only one commercial product (Grafix®) is a cryopreserved non-decellularized hC matrix [117,118].

There has been more interest around hC as it is richer in growth factors and cytokines than the amnion [119]. Here the authors determined the relative cytokine contribution of the hAM and hC in amniotic allografts. The levels of 18 cytokines involved in wound healing were measured in samples of PURION(R) processed dehydrated hAM, hC, and hACM grafts by multiplex enzyme-linked immunosorbent assay array. They found the amounts of each growth factor are practically consistent between hAM and hC. However, since hC is four to five times thicker than hAM, it contains substantially more of these factors per square centimeter than the amnion. They concluded that, when hAM is combined with hC to form a graft—as in dehydrated hACM or EpiFix—the resulting bilayer graft contains five times more growth factors than a single layer graft containing only the amnion. This suggests that dehydrated hACM may be more effective at delivering growth factors to a healing wound than amnion alone. Similarly, by subjecting the allografts to dehydration and electron beam irradiation or freezing processes, Kim et at. reported significantly higher levels of total cytokines in hACM than in hAM [120].

One study has shown that hC can be successfully decellularized and the main extracellular matrix proteins preserved [121]. Decellularized hC has two different surfaces: the reticular layer side and the trophoblast side. Both ultimate tensile strength and Young's modulus were significantly higher in decellularized hC compared to native tissue (hC stored at −80 °C) (p < 0.0001).

#### 1.2.2 Human umbilical cord

hUC represents the link between the mother and the fetus during a pregnancy, as it connects the developing embryo or fetus to the placenta. Because hUC-WJ—a part of hUC—is classified as a connective tissue, it is abundant in extracellular matrix, which is composed of glycosaminoglycans (mainly hyaluronic acid) and different types of collagens. Contrary to other species, such as pig or horse, WJ in humans lacks neural tissue, as well as other blood or lymph vessels [122].

There is increasing interest in using hUC-WJ as an allograft for soft tissue healing [25,123]. The collagen fibers present in hUC-WJ are comparable to the extracellular matrix of human articular cartilage, tendons, and dermal tissues [124]. Numerous clinical reports emphasize the regenerative potential of devitalized hUC-WJ for skin defects associated with bone exposure in fragile patients with underlying conditions (i.e., diabetes, ischemia, and osteomyelitis) [125]. Treatment using devitalized hUC-WJ matrix is associated with an overall healing rate of 79 %–100 % versus 32 % with standard of care. When hUC-WJ was applied to the sacroiliac joint or knee, there were statistically significant improvements in function, joint mobility, and pain relief [126,127]. These positive clinical results are mainly attributed to the presence of growth factors (HGF, VEGF and TGF-b), peptides and cytokines in clinically relevant quantities compared with other biological tissues [125,[128], [129], [130]]. *In vitro*, several growth factors have been found in the extracts of hUC-WJ; however, some assays required specific extracting solvents. As an example, 0.15 M acetic acid was a good extracting solution for TGF-β and PDGF (AB), while aFGF, bFGF and EGF were better extracted with 0.15 M Tris–HCl buffer at pH 7.6. The efficient extraction of IGF-I required 1 M acetic acid [131]. While hUC-WJ has been successfully applied in humans in over 180 different homologous use sites [132], the exact localization of hUC-WJ was not specified, as they were referred to as “hUC”.

hUC-WJ stroma is divided into three zones, namely, the sub-amniotic zone, the WJ, and the adventitia of blood vessels, even though the typical adventitia is not present in umbilical vessels [122]. Although cells extracted from these regions meet the criteria for being MSCs, each part of the hUC-WJ may contain a MSC population that differs from that of the other parts. Currently, it seems impossible to draw a conclusion about MSC heterogeneity based on the available data, which was obtained by very different protocols.

Like hAM, several formats of hUC-WJ are already commercialized: cryopreserved (CryoText™), air-dried (EpiCord, SecreText™, and ProText™). The PURION® PLUS Processed UC tissue (dHUC, EpiCord, MiMedx Group, Inc.) is processed using similar technology to PURION® Processed dehydrated hACM (dHACM; EpiFix/AmnioFix, MiMedx Group, Inc.) [133]. EpiCord matrix has biological properties that stimulate cellular responses for soft tissue healing. *In vivo* studies have shown that the injection of hUC-WJ prevents the joint alterations associated with arthritis progression [134]. In a retrospective study, patients reported less pain after injecting hUC-WJ (SecreText™) in rotator cuff defects [132].

The interest in hUC-AM is also increasing with two commercialized products: AmnioGuard® (Bio-Tissue, Miami, FL) [135] and Sclerfix® (Horus) [109]. However, the UC origin of hAM for the AmnioGuard® product is not clear (https://biotissue.com/products/ocular/amnioguard/). While other hUC-AM products are mentioned in Torre's review [7], the UC origin of these products is not clear when looking at the website of each company. Additionally, very few clinical [135] or preclinical [109] studies have been published. This allograft is primarily used in the field of ophthalmology, where we have obtained very good clinical results in our practice. The distinctive physical and biochemical attributes of hUC-AM make it a promising candidate for use in ocular surface reconstruction as a scleral or corneal substitute, for treating deep ulcers and corneal perforations or for fornix reconstruction. The advantage of hUC-AM grafting over hAM transplantation alone is that it has prolonged adherence without dissolution, thus allowing sufficient time for corneal healing; it also minimizes inflammation and enhances epithelialization. Furthermore, the hUC-AM has remarkable resilience to twisting and compression, such as that induced by eyelid blinking [136,137].

#### 1.2.3 Immunogenicity, host response and degradation

Like other authors, we hypothesized that PnD's immune privilege can be attributed to a lack of immunogenic molecules but also to the presence of immunomodulatory molecules including members of the B7 family of immunomodulatory cell-associated proteins, inhibin, activin, and follistatin [138].

Regarding the low immunogenicity of perinatal tissue, as reported above for hAM-derived cells, human chorionic plate (hCP)-MSCs also express HLA-G, which is implicated in their immunomodulatory effects (for references: [1]). Additionally, these cells have higher CD106 expression than MSCs derived from hUC, hAM, and decidua parietalis. hUC-WJ-MSCs express MHC class I molecules (classical HLA-A, -B, and –C, and non-classical HLA-G, -E, and -F) and lack HLA-DR (for references: [1]).

Within the COST Sprint, we have extensively studied the effects of PnD on the innate and adaptative immune response [31]. Additionally, randomized clinical trials over the past 10 years have evaluated the safety profile of placental-derived biomaterials and concluded that placental-derived biomaterials are safe when treating hard-to-heal ulcers [123].

Looking at each tissue more in detail, decellularized hC is biocompatible both *in vitro* and *in vivo* [121]. Notably, *in vivo* experiments revealed that by day 28, the decellularized hC began integrating with the host tissue. From our review of the literature, we found that deepithelialized and dehydrated hACM BioXclude® resorbs in 8–12 weeks (according to the manufacturer) but we were unable to find any proof of concept studies [22]. hUC-WJ is a less studied perinatal tissue. Based on *in vitro* studies, we showed that decellularized hUC-WJ is a biocompatible and bioactive matrix that supports the proliferation of primary cultured gingiva fibroblasts (Fibro) and alveolar osteoblast (Osteo) recruitment, while devitalized hUC-WJ had an effect on Fibro and Osteo recruitment [28]. When decellularized hUC-WJ was implanted subcutaneously in immunocompetent rats, the graft was totally resorbed after 3 weeks without any signs of foreign body reaction. No calcification and tissue vascularization were observed [28].

Interestingly, some authors postulated that decellularized hUC-WJ would provide a 3D environment that is well-suited to supporting the culture of undifferentiated MSCs [139]. When seeded onto decellularized hUC-WJ, hUC-WJ-MSC successfully attached to, and penetrated, the porous matrix, resulting in a slower rate of cell proliferation. When decellularized hUC-WJ was implanted into a murine calvaria defect model with green fluorescent protein labeled osteocytes, the osteocytes migrated into the matrix within 24 h. Importantly, this biocompatible matrix was still visible up to 14 days after transplantation.

#### 1.2.4 Processing and regulation

Within the COST SPRINT, a lack of PnD standardization has identified: from preparation to *in vitro* testing—an issue that may ultimately delay clinical translation [140]. So, a PnD e-questionnaire (https://www.frontiersin.org/articles/10.3389/fbioe.2023.1258753/, full#supplementary-material) has been developed to assess the current state of the art for the procurement, isolation, culturing, preservation and characterization of PnD *in vitro*. Furthermore, a consensus has been proposed for the scientific community on the minimum criteria that should be reported to facilitate standardization, reproducibility and transparency of data in PnD research.

Surprising, looking at the literature, most new perinatal tissues are not decellularized nor devitalized [7,9,13,22,81,115,116]. However, this step may ensure its safety and facilitate its approval by the national health authority and/or accelerate its regulatory course as it does not hold living cells.

Furthermore, hC, hACM and hUC-WJ mainly undergo a lyophilization or dehydration process, given the advantages described above. Here, like for hAM, there is often a confusion between lyophilization or dehydration processes that do not guarantee cell survival, and decellularization processes that fully remove DNA content. We also found minimal information on the processes applied to perinatal tissues on most tissue bank websites. In this case, no therapeutic bioactivity from cells is expected, because the matrix contains active biomolecules, as shown on hUC [28] and as suggested for cryopreserved hAM in our previous opinion paper [86]. Focusing on matrix and/or biomolecule roles where cell involvement is not required, has allowed the development of new scaffolds from PnD. For example, hAM extracts as well as modified fetal membranes have become commercially available as mechanically micronized or injectable tissue scaffolds [25,115].

Inside the COST SPRINT community, we revised and compared existing EU national regulations [34]. We provided an overview of the various options available to introduce PnD into clinical practice including: (1) the type of PnD, (2) the amount of available data, (3) the degree of manipulation, and (4) the intended application and path towards possible commercialization. Then, in order to facilitate the creation and potential commercialization of perinatal bioengineered and advanced pharmaceutical products and technologies, we described the necessary data collection and provided recommendations [35].

Regarding European regulations, in cases of homologous use, hC, hACM, hUC-AM, hUC-V or hUC-WJ products fall under the Regulation of Human Tissues and Cells (Directive 2004/23/EC), which has now been replaced by the new Regulation on Standards of Quality and Safety for SoHO [105]. As mentioned above for hAM, depending on the final mechanisms of action of non-viable SoHO or their derivatives, the new perinatal tissues fall within Regulation (EU) 2024/1938 [105] or Regulation (EU) 2017/745 ^106^. Here again, their full decellularization or devitalization is not confirmed. Additionally, these new allografts—except for hACM—are currently not included in the “Guide to the Quality and Safety of Tissues and Cells for Human Application” (5th Edition) [104]. The question we have previously raised about the decellularization/devitalization process not being a non-substantial modification in Europe [107] compared to FDA regulations [108] is also relevant.

In the USA, perinatal tissues are processed from human tissue according to American Association of Tissue Banks (AATB) standards and are regulated as a Human Cell, Tissue, or Cellular or Tissue-Based Product (HCT/P) by the FDA under section 361 of the Public Health Service Act (21 CFR, Part 127.10a) [108].

The massive influx of new PnD on the market forced the FDA to communicate specifically on this subject [141]. Thus, some readjustments appear necessary, in particular, a position statement about the need to apply a decellularization/devitalization process. Recently, the Perinatal Stem Cell Society has produced a position paper on the Regulatory Landscape for Perinatal Stem Cell and Tissue Products [142]. They noted that in Japan, regenerative medicine products are classified by official Regenerative Medicine Committees that are comprised of experts in the field. Consequently, this Committee classifies the products as either Class I (High Risk), Class II (Medium Risk) or Class III (Low Risk) regenerative medicine products. The Perinatal Society has suggested the U.S. adopt a similar classification system for stem cells and manipulated or conditioned tissue products. For example, by implementing the Japanese framework into the current U.S. regulatory framework, Section 361 HCT/Ps would be considered Class III Low Risk, which seems appropriate for minimally manipulated perinatal tissue products, as long as they meet the requirements outlined in Section 1271.10: hAM, hUC, hUC-WJ and placental tissue.

### 1.3 Perinatal tissues in bone repair

#### 1.3.1 Human amniotic membrane

In 2021, we did a systematic review to assess the benefits of using hAM and amniotic membrane-derived products for bone regeneration [14]. The first *in vivo* study was published in 2001 and the first human study in 2009. Among the 21 pre-clinical studies investigating the effectiveness of hAM for bone regeneration, two studies assessed its osteoinductive potential using an ectopic bone formation model (i.e., subcutaneous implantation), while all the remaining studies were conducted on orthotopic models. Most of the included studies assessed hAM, whereas two animal studies were performed with AM derived from rabbit or dog placenta. hAM was either applied over the bone defect or implanted as a filler inside the bone defect (like a bone substitute). The hAM format was fresh (n = 5) or preserved with different methods such as cryopreservation (n = 8), lyophilization (n = 3), de-epithelialization (n = 2), and decellularization (n = 7). Finally, hAM was mainly used alone or in combination with a bone substitute (n = 13). In some studies, hAM was seeded with MSCs before being implanted into animal models (n = 8).

Our review [14] also revealed the osteogenic potential of hAM in seven clinical studies, all performed in oral and maxillofacial surgery. hAM was used as an allograft membrane in a lyophilized format in six cases and decellularized then lyophilized in one. It was used to cover a bone substitute in six studies. A more recent review of literature [143] did not highlight any novel findings. We confirm that hAM is still currently solely used in oral and maxillofacial surgery as a barrier membrane combined with bone substitutes in guided bone regeneration (GBR) or guided tissue regeneration (GTR) [144,145].

#### 1.3.2 Human chorion and amnio-chorionic membrane

Similar to hAM, hC and hACM are also used as barrier membranes in combination with other materials and are not used alone in bone regeneration applications.

In this context, we did a systematic review of the clinical applications in which hC and hACM were used for oral tissue regeneration procedures [13]. The first use was reported in 2015. Among the 21 included articles, the hACM and hC were in a dehydrated format—mostly preceded by de-epithelialization (Purion process)—in 11 studies and a freeze-dried format in eight studies, preceded by decellularization in one study. Both freeze-dried and dehydrated membranes were subsequently sterilized by gamma-radiation. Nine studies used commercially available BioXclude ACM, a minimally manipulated dehydrated de-epithelialized ACM. For bone repair indications in oral surgery (intrabony and furcation defect treatment), CM and ACM were often combined with bone substitutes. There is no evidence that hC and hACM can improve bone repair when used alone.

A more recent review [146] confirmed its exponentially growing use in the oral surgery field. We confirm its extensive use mainly as GBR membranes combined with bone substitutes [[147], [148], [149], [150], [151], [152], [153], [154]] or biodegradable natural polymers [155].

Recently, two studies have reported interesting results beyond GBR applications. Chopra et al. compared hACM and platelet-rich fibrin with no additional bone substitutes on new bone formation and soft tissue healing in extraction sockets indicated for rehabilitation with dental implants [156]. They showed that most of the sites had a larger amount of bone formation with hACM, which confirms its osteogenic potential. However, the difference was not statistically significant when compared with platelet-rich fibrin. Tacktill et al. applied a dehydrated hACM (Salera Allograft Placental Membrane, MTF Biologics, Edison, New Jersey) directly to the periosteal layer to assist bone healing in one case of reconstructive midfoot surgery and one case of pilon fracture/ankle [157].

#### 1.3.3 Human umbilical cord and Wharton's jelly

A few *in vitro* and *in vivo* studies have investigated the osteogenic potential of hUC-WJ in bone repair. Yu-Show Fu et al., prepared two types of hUC-WJ: cryopreserved using cryoprotective agents; lyophilized and decellularized. They found that hUC-WJ can promote cell adhesion, differentiation, and maturation of rat osteoblasts *in vitro*, and also help bone regeneration in rats with critical-sized calvaria defects [158].

In another study, hUC-WJ matrix was combined with bioceramic and bioglass particles to produce bone substitute. When introduced in rat tibial defects, the biocompatibility was high and good integration with the bone tissue was found by histological analysis [159]. Cleft lip and cleft palate are among the most common congenital birth defects. Cryopreserved hUC-WJ was implanted into a critical-size alveolar bone defect model. New bone growth was stimulated leading to partial-to-full closure of the defect in the animals treated with hUC-WJ (156 % ± 21 %) versus the control group (50 % ± 21 %) (*p* < 0.05) [160,161].

Based on these results, Charles S. Cox, Jr., a Professor of Pediatric Surgery, proposed treating cleft lip and cleft palate using autologous hUC-WJ [160,161]. The majority of infants with cleft lip and palate are diagnosed during routine ultrasound exams around 24 weeks of gestation. Traditionally, surgeons have repaired alveolar cleft palates with a bone graft from the iliac crest of the pelvis. However, this subjects the child to an additional surgical procedure with associated discomfort and risks. An ultrasound diagnosis enables the parents and doctors to plan and prepare for cleft repair after birth. If the repair will incorporate hUC-WJ to augment surgery, the parents know in advance that they need to arrange for its collection and storage. However, large animal preclinical studies and FDA approval are required prior to translating this approach to treat children with cleft lip and palate.

As far as we know, hUC-AM was not investigated in bone repair.

### 1.4 Our experience with perinatal tissues as GBR membranes

We developed our own decellularization and lyophilization process and investigated the resulting hAM/hACM as well as hUC-WJ prepared as a membrane in GBR applications [17,18,20] (Graphical abstract and Table 1 (A and B)).

**Table 1 (A and B):** Our own process applied to perinatal tissues with their advantages and drawbacks (adapted from Refs. [[17], [18], [21], [28], [29], [30], [165]]). Abbreviations: hAM: human amniotic membrane, hACM: human amnio-chorionic membrane, hUC-WJ: human umbilical cord Wharton’s jelly.

**A**			
	Decellularized hAM	Decellularized and lyophilized hAM	Decellularized and lyophilized hACM
Full decellularization	–	+	+
Lyophilization	+	+	+
Gamma sterilization	+	+	+
Advantages	•Room temperature storage •Immediate use (no need to order tissue and thaw it) •Cutting is possible •Ease of handling •Improved mechanical properties •Removal of cellular components that could trigger immunological response		
	Faster processing than decellularized products	•Decreased degradation rate •More suitable scaffold for cell proliferation and osteodifferentiation	•Increased thickness compared to hAM and decreased degradation •Not proven yet but expected to be rich in growth factors due to chorion
Drawbacks	Lack of cellular components that could have positive impact		
	•Fast degradation compared to Decellularized and lyophilized hAM •Remaining immunological component •Difficult to suture	Difficult to suture	Increased duration of decellularization
Article reference	[18,21]		[164]

**B**		
	Devitalized and decellularized hUC-WJ	Devitalized hUC-WJ
Devitalization	+	+
Full decellularization	+	–
Lyophilization	+	+
Advantages	•Immediate use after gamma sterilization •Remove of cellular components that could trigger immunological response •Increase in tissue porosity •Increase in tissue bioactivity	
	•Antibacterial effect ++ •Proliferation of primary cultured gingiva fibroblasts and alveolar osteoblast recruitment	•Antibacterial effect + •Primary cultured gingiva fibroblasts and alveolar osteoblast recruitment only
Drawbacks	• Lack of standardized protocol for tissue preparation •Rapid degradation •Lack of cellular components that could have positive impact	
Article reference	[28,29]	

+ = yes; − = no; ++ = high.

The hAM was decellularized using an enzymatic method followed by a detergent decellularization method [18]. The results of this process were determined using DNA staining and quantification. Some samples were frozen at −80 °C, then dried under vacuum in a freeze dryer and gamma-sterilized [18].

Different conditions were tested in two animal models to assess the hAM's osteogenic capacity. In brief, we first demonstrated that hAM extracellular matrix plays a greater role than hAM cells during GBR [17,18,20]. Secondly, decellularized and lyophilized hAM was successfully used to reduce the two-step surgery called “induced membrane technique” or “Masquelet technique” to a one-step surgery when treating long bone defects or non-union fractures [12,162,163]. One explanation for these successful results was that this hAM format had the slowest rate of resorption compared to fresh, cryopreserved, or lyophilized-only hAM. It was the only membrane still present 8 weeks after its subcutaneous implantation in rats [21]. From this study, we concluded that the slow degradation of decellularized and lyophilized hAM was more suitable for bone tissue repair than the other hAM formats because of the time required to obtain fully mature bone. The second explanation was that the hAM decellularization process increased its ability to support host cell adhesion and proliferation.

However, hAM has a poor ability to maintain space, which is often necessary for GBR applications. We hypothesized that keeping the amnion attached to the hC may improve its mechanical properties. We have therefore adapted the decellularization and lyophilization process to hACM and are currently evaluating its mechanical properties and ability to act as a barrier membrane for GBR compared to hAM alone [164].

The hUC-WJ, when prepared as a membrane, underwent a decellularization process following devitalization via two freeze-thaw cycles (−20 °C/20 °C). This process involved treatment with Triton and DNase, after which the tissue was frozen at −20 °C and then −80 °C before undergoing freeze-drying [[26], [27], [28], [29]]. *In vitro*, the devitalized and decellularized hUC-WJ was found to have an immunomodulatory effect, reducing the up-activation of circulating polynuclear neutrophils and monocytes. Macrophages in contact with this processed tissue express a hybrid M1/M2 phenotype. *In vivo*, limited regeneration of bone at the marginal area of the calvaria defect was observed. Indeed, the decellularized hUC-WJ prepared as a membrane had induced osteolysis [[26], [27], [28], [29]]. We speculated that the immunomodulatory effect, the fast degradation rate as well as the poor mechanical properties make the hUC-WJ prepared as a membrane unsuitable for repairing hard tissues like bone. The osteocompatibility of decellularized hUC-WJ was improved through collagen crosslink strategies using Genipin [30] and tannic acid [165]). The degradation rate and mechanical properties of crosslinked hUC-WJ with covalent bounds were greatly improved. *In vivo*, cross-linked hUC-WJ increased newly formed bone in critical calvaria rat model.

### 1.5 Our experience with perinatal tissues as a bone substitute

In our review of the literature, we found that some authors investigated the use of hAM as a bone substitute to fill bone defects [14]. Poor or delayed regeneration was observed, thereby suggesting that filling a bone defect with hAM hinders the bone regeneration process. Potential explanations are the poor colonization capacity of amniotic cells when cryopreserved or when fresh hAM is inserted in the defect, the lack of vascularization in the hAM, and the insufficient mechanical properties of hAM as a bone void filler. It seems that hAM is not an appropriate scaffold to support bone formation and ensure bone growth. Still, it could encourage bone repair by promoting mineralization [65] or by attracting osteoprogenitor cells [166]. Our own experiments did not yield conclusive results (unpublished observations).

To our knowledge, hUC-WJ was never used as bone void filler.

## 2 Discussion/conclusion

This opinion paper aims to provide our insights on decellularized or devitalized-lyophilized human perinatal tissues for bone repair. We summarized several aspects, including immunogenicity and resorption, lyophilization/dehydration/decellularization and devitalization processes, regulation, and bone repair applications.

If we look back on the historical work conducted on hAM, its immunogenicity and antigenicity have only been described sporadically. Therefore, despite good clinical outcomes, we believe that stronger evidence is needed. In this context, we did a clinical pilot study in which cutting-edge technologies were applied to answer these questions [167].

hAM immunogenicity is closely linked to the presence of viable cells or cell/DNA debris. The evaluation of viable residual cells in cryopreserved hAM is not currently done during the banking process or during *in vivo* or clinical studies. This is due to its high tolerance. Additionally, there is no definitive evidence about the role of endogenous amniotic cells from cryopreserved hAM in tissue repair. Furthermore, cellular capacity varies across the anatomical subregions of hAM, a factor that has not yet been considered in tissue banking procedures due to its high complexity.

As there have often been questions about residual viable cells in hAM, decellularization or devitalization processes have been developed to enhance hAM tolerance/safety. From a regulatory perspective, these processes do not qualify as non-substantial modifications. Furthermore, non-viable or rendered non-viable SoHo may fall under Regulation (EU) 2024/1938 [105] or Regulation (EU) 2017/745 [106]. However, neither recommendation specifies how the tissue should be rendered non-viable. Consequently, no standard protocols for decellularization or devitalization, nor related controls, have been described.

Additional sterilization by gamma-radiation also improves its safety. To expand hAM use, there has been growing interest in the lyophilization or dehydration process, which facilitates its handling, cutting, and storage in the operating room while also reducing degradation.

All these processes have a significant impact on biomechanical properties, thickness, degradation, and biocompatibility [73,101]. However, due to inter-donor [168] and intra-donor variability [66], it is very complicated to substantiate these effects. We recognize that lyophilization or devitalization processes impact cell viability but leave residual DNA content, cell-associated proteins, and other cellular debris that are not being described sufficiently.

Second, in the last decade, all the technologies developed for hAM have been applied to new perinatal tissues such as hC, hACM, hUC, hUC-AM, hUC-V, or hUC-WJ. These new allograft formats are widely associated with patented technologies and most of them are commercialized [81]. Their regulatory classification is subject to the same questions as hAM about the decellularization or devitalization definition in the applicable regulation. We noted that hC, hACM and hUC-WJ are primarily used for tissue healing support and not used in ophthalmology applications, contrary to hAM (fetal membrane origin).

Like for hAM, these processes impact the characteristics of perinatal tissues [101,123], but it has been challenging to confirm them due to donor variability [169,170]. Most commercial products are lyophilized or dehydrated, with no information provided on residual DNA content, cell-associated proteins, or other cellular debris. It appears that these products are often presumed to be decellularized solely due to the lyophilization/dehydration process. Consequently, we suggest tissue banks provide more detailed descriptions of their products on their website, particularly regarding the use of decellularization or devitalization processes and the subsequent testing for residual cellular debris/DNA.

Like others, we are considering how to incorporate these new perinatal tissues in our therapeutic strategy, focusing on their predominant matrix function and growth factor/cytokine content. As an example, we are particularly interested in hACM given its greater thickness and higher growth factor and cytokine content in the chorion than in hAM alone. Additionally, hUC-WJ releases antimicrobial agents that limit the growth and adhesion of Gram-positive bacteria (S. aureus and S. epidermidis) [28]. Our experiments have shown that the decellularization process enhances the efficiency of lyophilized hAM, hACM, and hUC-WJ.

Finally, our literature analysis revealed that the use of hAM in bone repair remains challenging, despite its initial use in animal studies in the early 2000s and in humans approximately a decade later. We confirmed these observations through *in vivo* studies. This limitation is mainly due to the lack of both inherent osteoconductive and angiogenetic properties combined with—for cryopreserved hAM—the questionable role of residual viable cells. Recent research has introduced a decellularized placental sponge derived from fetal membranes, demonstrating its potential as an excellent anti-inflammatory 3D substrate for both the culture and delivery of autograft or allograft MSCs into bone defect sites, ultimately facilitating improved bone healing *in vivo* [171].

Our experiments confirmed previous findings indicating that, to date, hAM appears to be best used as a GBR or GTR membrane in combination with bone substitutes for applications in oral and maxillofacial surgery. In reviewing the literature, platelet-rich fibrin and platelet-rich plasma may be considered alternative materials to hAM in GBR or GTR procedures. However, a comprehensive literature review indicates limited evidence supporting this comparison [172]. hAM has been successfully used in the management of osteonecrosis, compared to standard procedures [173]. Following a successful compassionate use case in the same indication [23], we initiated a multicenter randomized clinical trial (NCT05664815). If the results are encouraging, this could lead to expanded authorization for hAM for this indication in France.

Due to higher growth factor content and greater thickness, hC and hACM safeguarded by a decellularization process are increasingly being used in oral surgery. From our experiments, like other authors, we were unable to demonstrate a significant beneficial role of hACM in bone repair when used alone. Again, their use in GBR/GTR procedures seems more fruitful.

Regarding hUC-WJ, the results reported in the literature are contradictory. Its role in bone repair and regeneration should be further studied in orthopedic applications. Like for the other perinatal tissues, it appears best suited to being used in GBR/GTR.

The primary focus of using hUC-AM has been on assisting soft tissue healing. As with the historical use of hAM (derived from fetal membranes), its current applications are primarily in ophthalmologic indications. Its potential for supporting bone repair, or at a minimum, in GBR/GTR procedures, remains unexplored and warrants further investigation.

Consequently, some challenges remain:

➢ The need to better distinguish between the concepts of “lyophilization/dehydration”, “full decellularization” and “devitalization”. And when a decellularization/devitalization process is applied, to document its impact on cell viability/vitality to support the non-viable or rendered non-viable SoHO definition.
➢ To integrate the high complexity when qualifying perinatal tissues due to both intra- and inter-donor variability and the impact of all the processing methods used (lyophilization, dehydration, decellularization, devitalization and gamma sterilization).
➢ The capacity of tissue banks to collect and store new perinatal tissues, especially when they have not implemented the required practices.
➢ The use of perinatal tissues as bioactive GBR/GTR membranes but with no cost-benefit analysis and limited conclusive randomized clinical trials.
➢ The restricted use of perinatal tissues as bone substitutes if they are used as such without additional engineering.
➢ The applicable regulatory requirements when these allografts are used in a non-homologous manner, as is the case for bone repair.

## CRediT authorship contribution statement

**Lauriana Solecki:** Writing – review & editing, Writing – original draft, Visualization, Validation, Methodology, Investigation, Formal analysis, Data curation, Conceptualization. **Mathilde Fenelon:** Writing – review & editing, Writing – original draft, Visualization, Formal analysis, Data curation, Conceptualization. **Halima Kerdjoudj:** Writing – review & editing, Writing – original draft, Visualization, Validation, Methodology, Investigation, Formal analysis, Data curation, Conceptualization. **Roberta Di Pietro:** Writing – original draft, Visualization, Validation, Methodology, Investigation, Formal analysis, Data curation. **Gianmarco Stati:** Validation, Methodology, Investigation, Data curation. **Camille Gaudet:** Methodology, Investigation, Data curation. **Eugenie Bertin:** Investigation, Formal analysis, Data curation. **Jeremie Nallet:** Methodology, Investigation, Data curation. **Aurélien Louvrier:** Methodology, Investigation, Data curation. **Thomas Gualdi:** Methodology, Resources, Validation. **Jessica Schiavi-Tritz:** Writing – review & editing, Writing – original draft, Visualization, Validation, Methodology, Investigation, Formal analysis, Data curation, Conceptualization. **Florelle Gindraux:** Writing – review & editing, Writing – original draft, Visualization, Validation, Supervision, Project administration, Methodology, Investigation, Formal analysis, Data curation, Conceptualization.

## Ethical approval

Not applicable.

## Informed patient

Not applicable.

## Funding

Not applicable.

## Declaration of competing interest

The authors declare that they have no known competing financial interests or personal relationships that could have appeared to influence the work reported in this paper.

## Data Availability

No data was used for the research described in the article.
